# Supplementary material for: Covalent Organic Frameworks-TpPa-1 as an Emerging Platform for Electrochemical Sensing
Source: Nanomaterials (Basel). 2022 Aug 26;12(17):2953. doi: 10.3390/nano12172953 (PMC9457582; doi:10.3390/nano12172953)
Supplement: Supplementary file 1 [file nanomaterials-12-02953-s001.zip › nanomaterials-1873146 - supplementary.pdf]

nanomaterials

Supplementary Materials

Covalent Organic Frameworks-TpPa-1 as an Emerging Platform for Electrochemical Sensing

Gang Li, Baiqing Yuan *, Sidi Chen, Liju Gan and Chunying Xu

School of Chemistry and Materials Science, Ludong University, Yantai 264025, China
* Correspondence: baiqingyuan1981@126.com or bqyuan@ldu.edu.cn

The electroactive surface areas of these electrodes could be calculated by the Randles–Sevcik equation using the CV responses of the corresponding electrodes in 0.1 M KCl solution in the presence of 5 mM $K_3Fe(CN)_6$ at different scan rates at 298 K (Hosseini, H., Ahmar, H., Dehghani, A., Bagheri, A., Tadjarodi, A., Fakhari, A.R., 2013. Biosens. Bioelectron. 42, 426-429.):

$$I_p = 2.69\times10^5\times A\times n^{3/2}\times D^{1/2}\times C\times \upsilon^{1/2}$$

where, I_p (A) is the peak current, A (cm^2) is the electroactive surface area, n is the number of electron transfer which equals 1, D = 7.6×10^{-6} (cm^2/s), υ is the scan rate (V/s), C is the concentration of $K_3Fe(CN)_6$ (mol/mL).

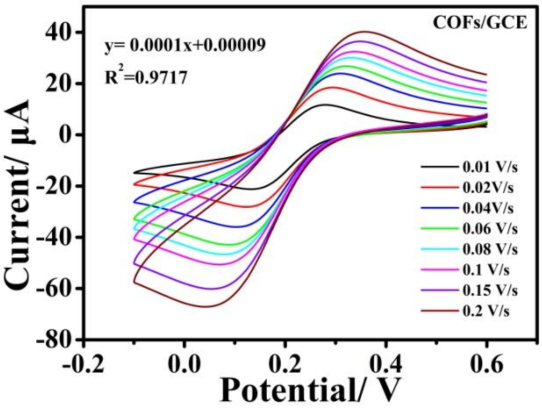

Figure S1. The CVs of COFs/GCE in 0.1 M KCl solution in the presence of 5 mM $K_3Fe(CN)_6$ at different scan rates.

COF/ GCE: A=0.027 cm^2

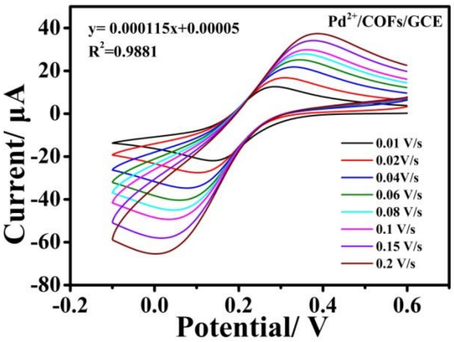

Figure S2. The CVs of Pd^{2+}/COFs/GCE in 0.1 M KCl solution in the presence of 5 mM $K_3Fe(CN)_6$ at different scan rates.

Pd/ COF/ GCE: A=0.041 cm²

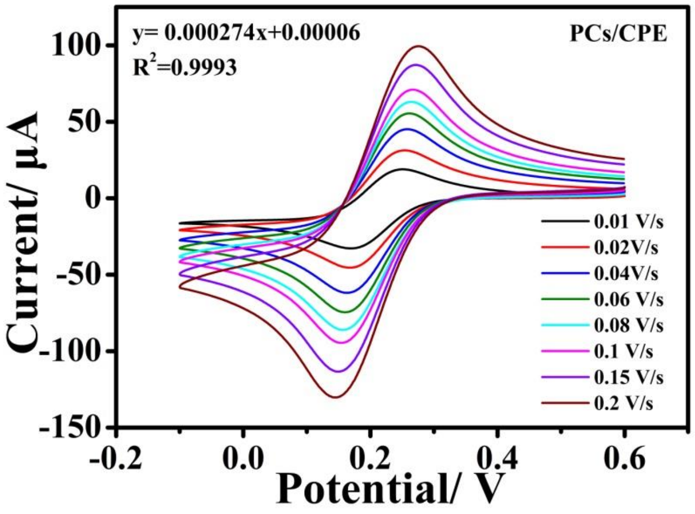

Figure S3. The CVs of PCs/GCE in 0.1 M KCl solution in the presence of 5 mM $K_3Fe(CN)_6$ at different scan rates.

PCs: A=0.074 cm²

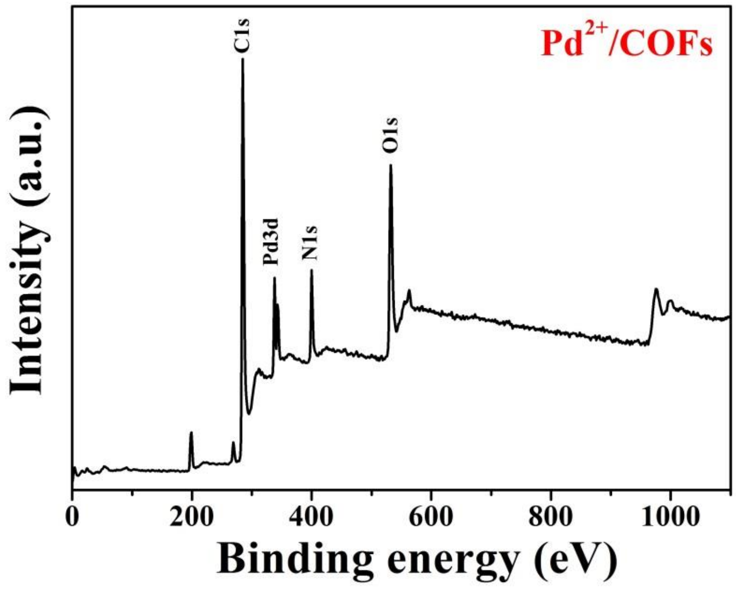

Figure S4. XPS spectra of Pd^{2+}/COFs.

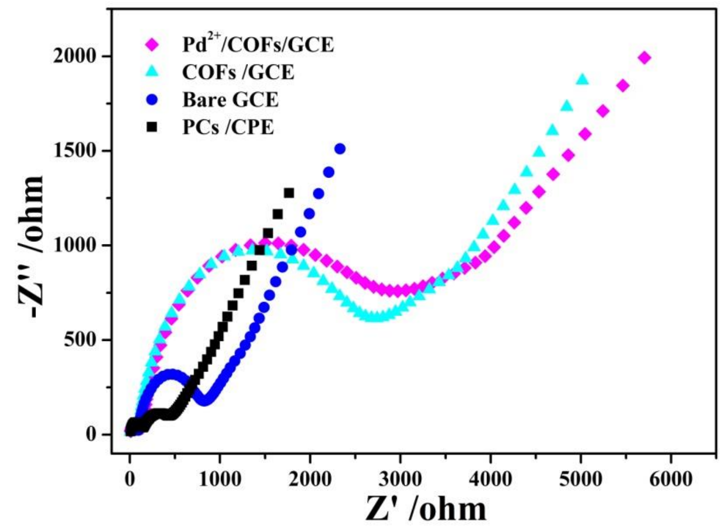

Figure S5. EIS of PCs /GCE, Bare GCE ,COFs/GCE, and Pd^{2+}/COFs/GCE in 0.1 M KCl solution in the presence of 5 mM $K_3Fe(CN)_6$.

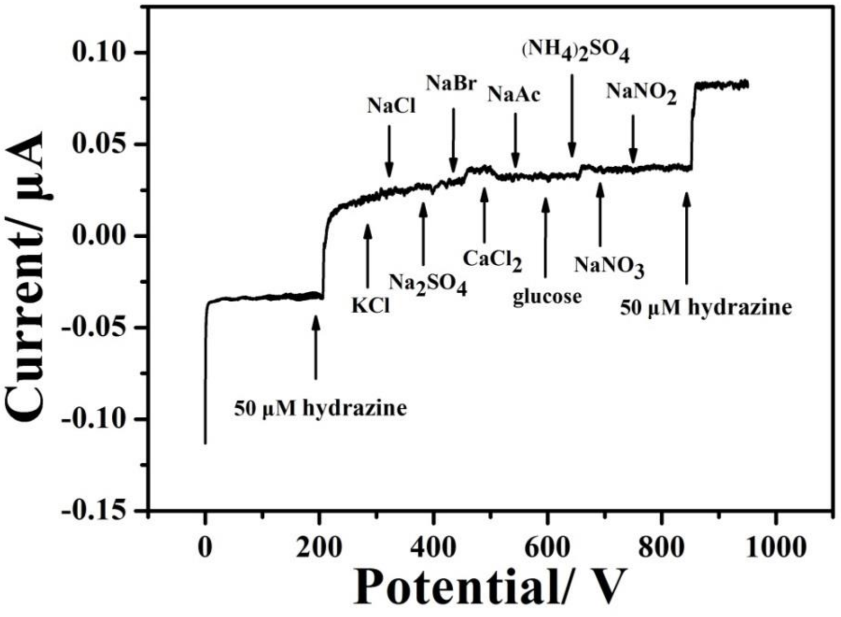

Figure S6. Amperometric curve of Pd^{2+}/COFs/GCE with 50 µM hydrazine and 500 µM of interferences such as KCl, NaCl, Na_2SO_4, NaBr, $CaCl_2$, NaAc, glucose, $(NH_4)_2SO_4$, $NaNO_3$, and $NaNO_2$. (Electrolyte: 0.1 M NaOH).

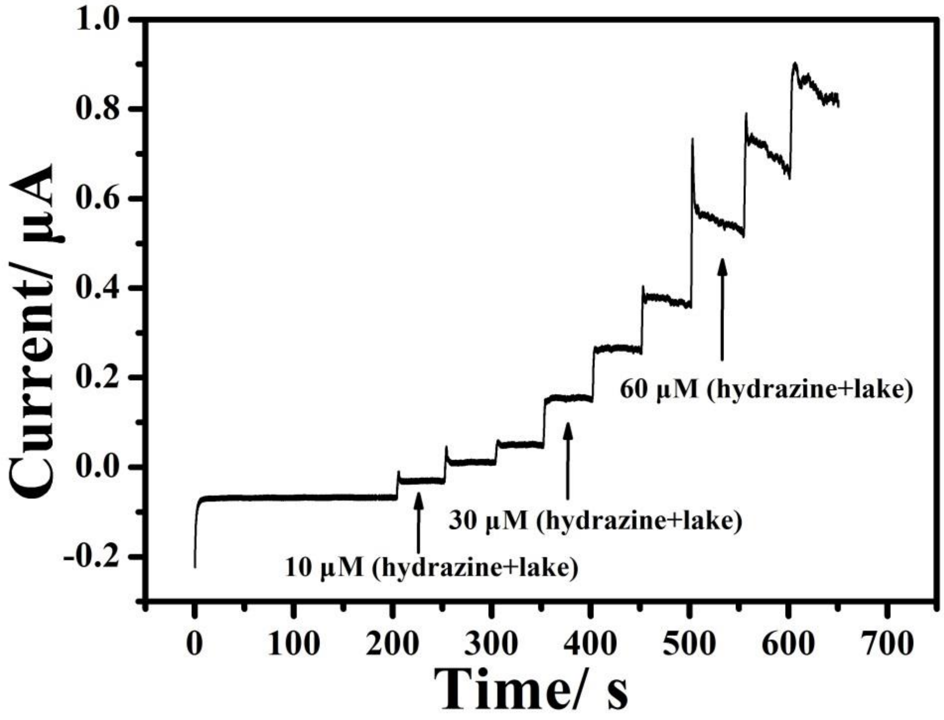

Figure S7. Amperometric curve of Pd^{2+}/COFs/GCE for the detection of hydrazine spiked in water samples at −0.1 V. (Electrolyte: 0.1 M NaOH).

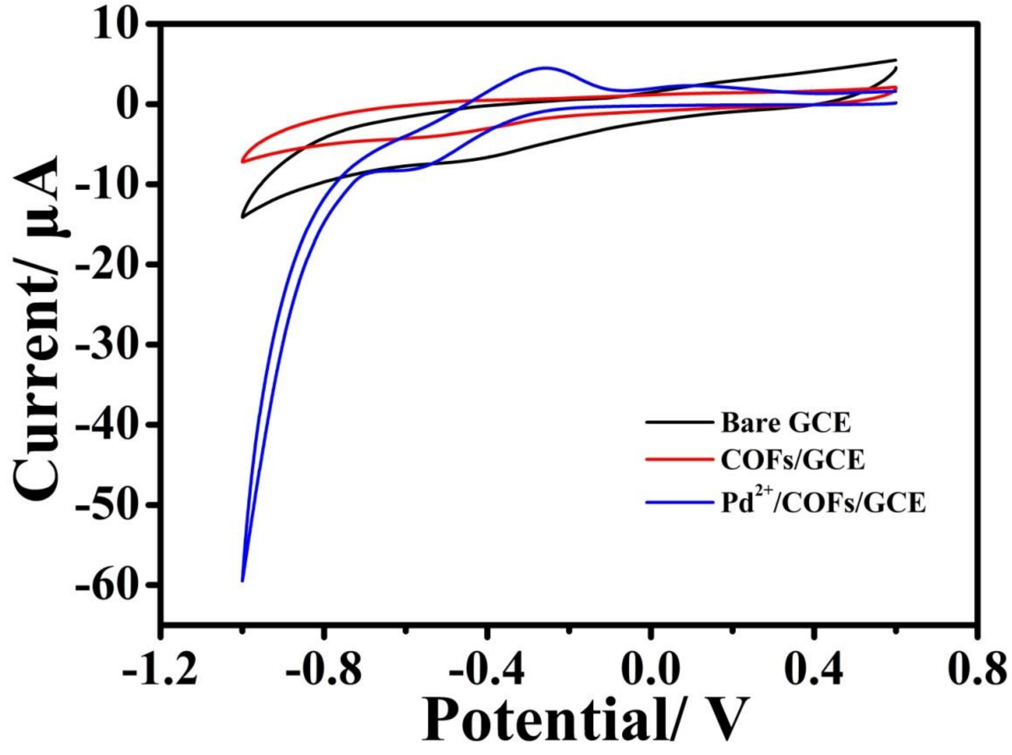

Figure S8. CVs of Bare GCE, COFs/GCE, and Pd^{2+}/COFs/GCE in 0.1 M PBS at a scan rate of 50 mV s^{-1} (pH=7.0).

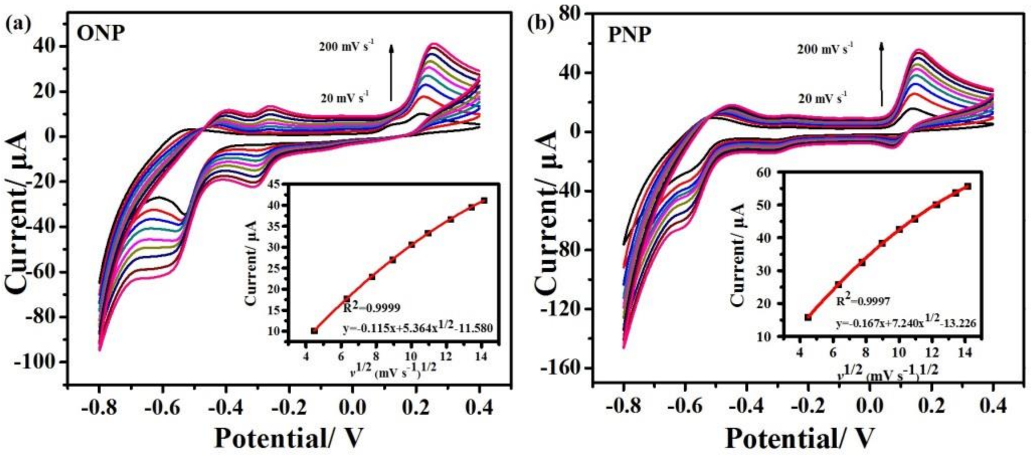

Figure S9. CVs of 500 μM of (**a**) ONP and (**b**) PNP on Pd^{2+}/COFs/GCE with different scan rates (20-200 mV s^{-1}) in 0.1 M PBS (PH=7.0). (Inset: the corresponding calibration plot.).

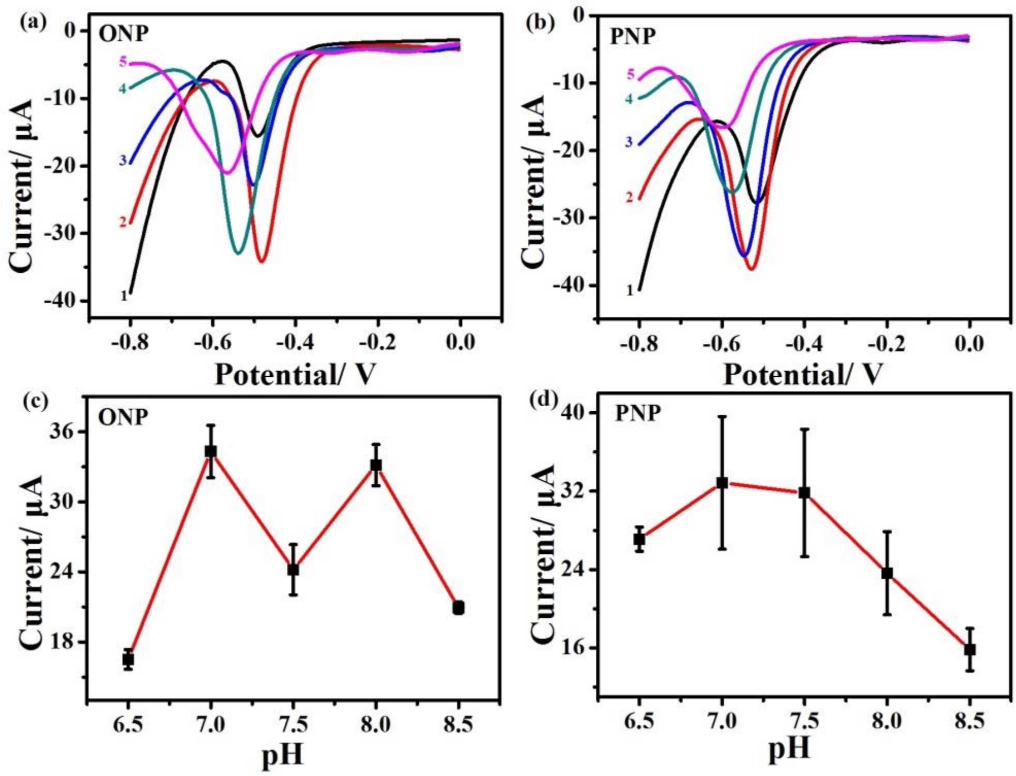

Figure S10. DPV curves of 500 μM of (**a**) ONP and (**b**) PNP on Pd^{2+}/COFs/GCE in 0.1 M PBS (pH=7.0) with different pH values (1-5: 6.5, 7.0, 7.5, 8.0, and 8.5). Plots of I_{pc} vs. pH for (**c**) ONP and (**d**) PNP.

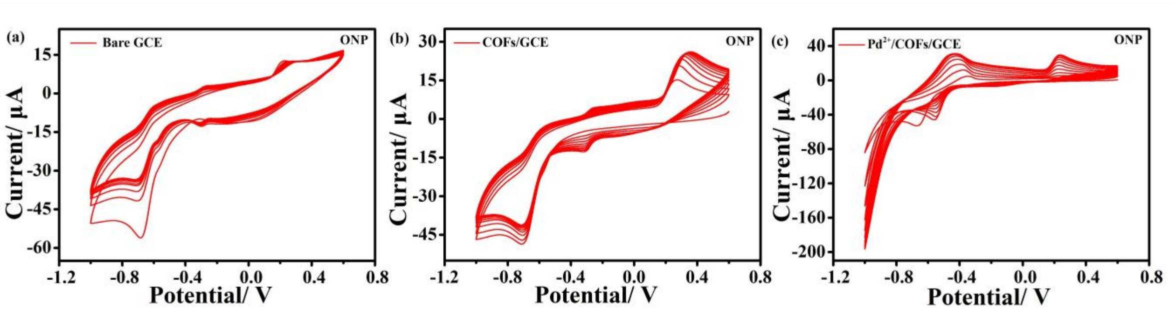

Figure S11. Successive CVs of Bare GCE (**a**), COFs/GCE (**b**), and Pd^{2+}/COFs/GCE (**c**) in the presence of 500 μM nitrophenol ONP at a scan rate of 50 mV s^{-1} in 0.1 M PBS (pH=7.

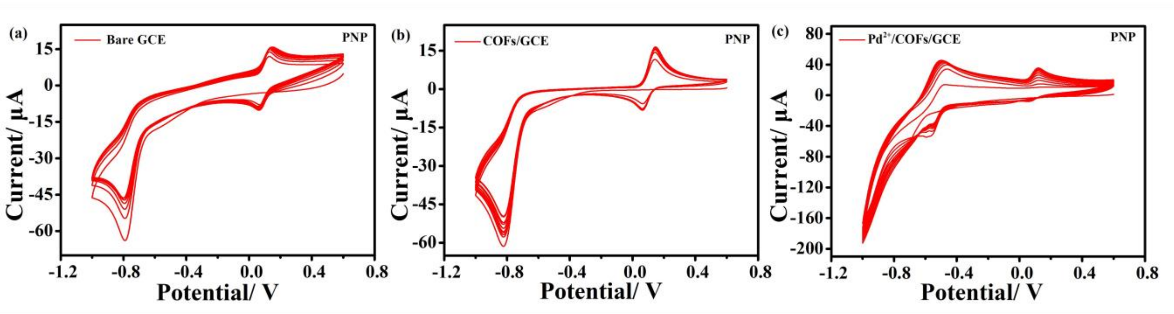

Figure S12. Successive CVs of Bare GCE (**a**), COFs/GCE (**b**), and Pd^{2+}/COFs/GCE (**c**) in the presence of 500 μM nitrophenol PNP at a scan rate of 50 mV s^{-1} in 0.1 M PBS (pH=7.0).

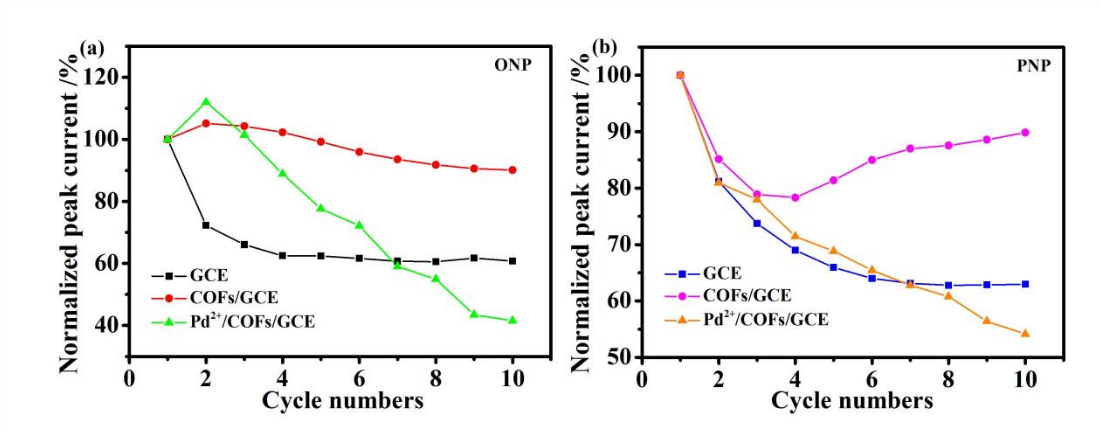

Figure S13. Normalized peak current of bare GCE , COFs/GCE , and Pd^{2+}/COFs/GCE in the presence of 500 μM nitrophenol ((**a**): ONP, (**b**): PNP) at a scan rate of 50 mV s^{-1} in 0.1 M PBS (pH=7.0) for ten cycles.

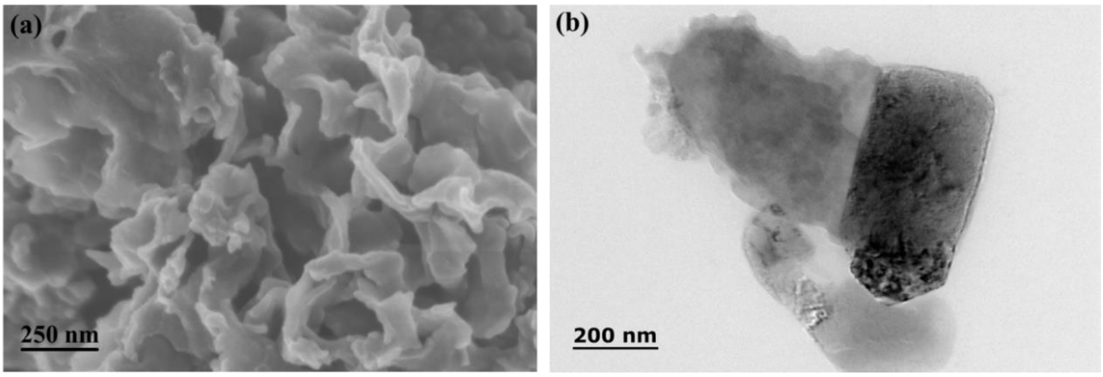

Figure S14. SEM (**a**) and TEM (**b**) images of PCs.

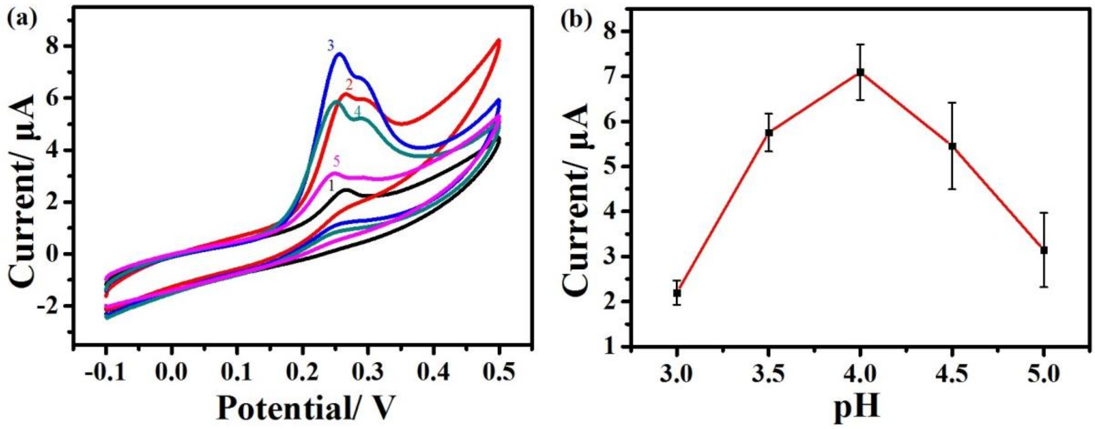

Figure S15. CVs of 5 mM of GSH on (**a**) PCs/CPE in 0.1 M PBS with different pH values (1-5: 3.0, 3.5, 4.0, 4.5 and 5.0). Plots of I_{pc} vs. pH for (**b**) GSH.

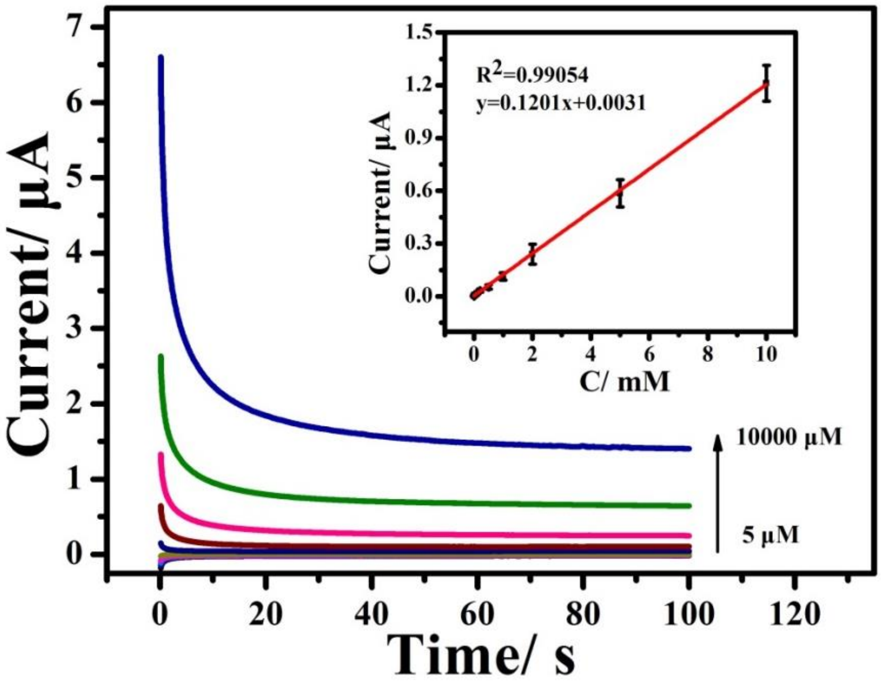

Figure S16. Amperometric responses of PCs/CPE to additions of various concentrations of GSH in 0.1 M PBS (pH=4.0) at 0.25 V. (Inset: the corresponding calibration plot.).

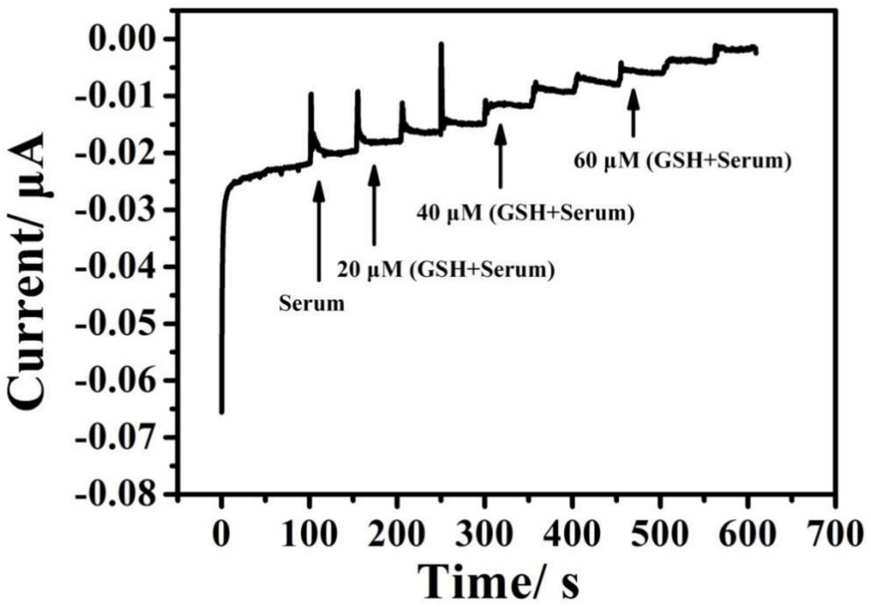

Figure S17. Amperometric responses of PCs/CPE to successive additions of various concentrations of GSH spiked in serum in 0.1 M PBS (pH=4.0) at 0.25 V.

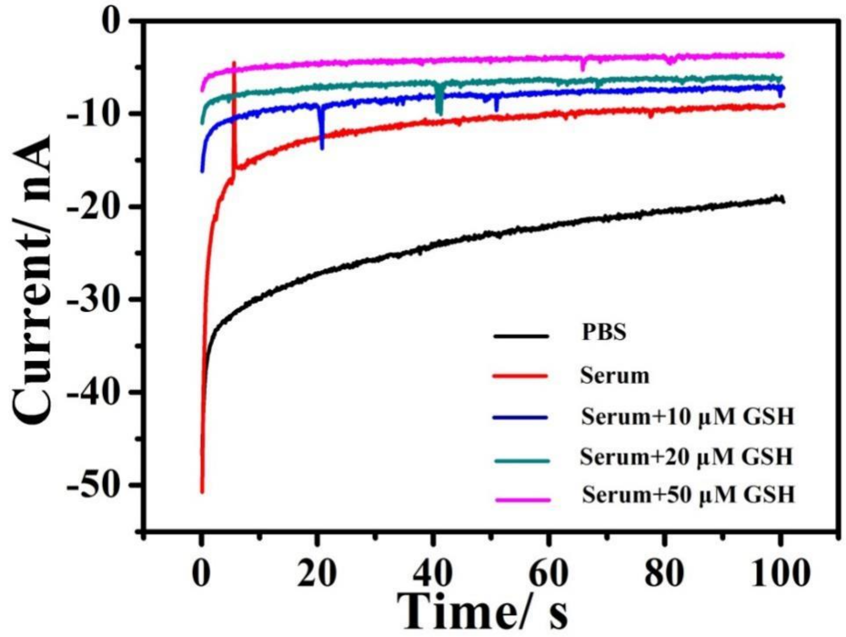

Figure S18. Amperometric responses of PCs/CPE for the detection of GSH spiked in human serum in 0.1 M PBS (pH=4.0) at 0.25 V.

Table S1. Comparison of the Pd^{2+}/COFs/GCE for different parameters with previously reported hydrazine electrodes.

Electrode Material	Electrochemical Method	Potential (V)	Linear Range(μM)	Detection Range (μM)	Ref.
Cu-MOFs/OMC/GCE	Amperometry	0.4	0.5-771	0.35	[1]
CuO/OMC/GCE	Amperometry	0.31	1-2110	0.887	[2]
MSRG/Au/GCE	Amperometry	0.29	2-30	0.5	[3]
MXene/ZIF-8/GCE	Amperometry	0.35	10-7700	5.1	[4]
Au@porous P-MWCNT/rGO/GCE	Amperometry	0.4	3000-55000	0.31	[5]
CNT/NiHCF/GCE	Amperometry	0.36	20-200	0.8	[6]
Cu NP/C	Amperometry	0.45	100-2400	1.4	[7]
Ag/ZIF-67/CPE	Amperometry	-0.05	4-326<br>326-4700	1.45	[8]
NPs/CPE	Amperometry	0.5	1-1300	0.28	[9]
$NiCo_2S_4$/GCE	Amperometry	0.3	1.7-7800	0.6	[10]
Pt-Pd/ERGO/GCE	Amperometry	-0.71	7-5500	1.7	[11]
PAYR/Ag@C/GCE	Amperometry	0.4	1-1320	0.25	[12]
rGO-Co_3O_4@Au/GCE	Amperometry	0.079	10-620	0.443	[13]
ZnO/Co3O4/GCE	Amperometry	-0.2	2-1500<br>1500-5500	0.52	[14]
**Pd^{2+}/COFs/GCE**	**Amperometry**	**-0.1**	**0.5-1588.5**	**0.2**	**This work**

Table S2. Determination of hydrazine spiked in water samples.

Added (μM)	Found (μM)	Recovery (%)	RSD (%)
10	9.5	95	7.3
30	26.7	89	3.9
60	51.3	85.3	1.4

Table S3. Comparison of different modified GCE for detection of ONP and PNP.

Electrode Material	Electrochemical Method	Linear Range (μM)		Detection Limit (μM)		References
		ONP	PNP	ONP	PNP	
BSO-gCN/GCE	LSV	/	1.6-50	/	1	[15]
AcSCD-AuNPs-MC/GCE	DPV	/	0.1-10<br>10-350	/	3.63	[16]
CeO_2:Ag/GCE	CV	/	7.81-1000	/	1.2	[17]
5-sulfosalicylic acid doped polyaniline/GCE	DPV	/	6.7-112.1	/	3.2	[18]
BaO NRs/GCE	DPV	5-640	/	0.5	/	[19]
Mg/Fe-LDH /GCE	Amperometry	1-700	/	4	/	[20]
SDS-HTLC/GCE	DPV	1-600	/	0.5	/	[21]
MnFe-PBA/GCE	DPV	1-700	/	0.59	/	[22]
nano-Au/GCE	CV	10-1000	10-1000	8	8	[23]
meso-$ZnCo_2O_4$/GCE	DPV	1-4000	1-4000	0.3	0.3	[24]
PEDOT:PSS/GCE	LSV	10-3000	10-3000	4.55	4.51	[25]
**Pd^{2+}/COFs/GCE**	**DPV**	**5-2000**	**5-2000**	**1.75**	**0.91**	**This work**

Table S4. Comparison of different modified electrodes for electrochemical sensing of GSH.

Electrode Material	Electrochemical Method	pH	Linear Range (μM)	Detection Limit (μM)	Ref.
GO/GCE	Amperometry	5	5-875<br>875-4080	5	[26]
CoPcS-NWE	Amperometry	4	10-20000	8.3	[27]
N-doped ZnO/C hollow rhombic dodecahedral/GCE	CV	7.4	1-500	8	[28]
MWCNTs/SPE	CV	7.3	5-20	2	[29]
CNTs/SPE	CV	7	10-60	3	[30]
Co-MOCP/CPE	Amperometry	5.5	2.5-950	2.5	[31]
Pt/rGMnO	Amperometry	6.5	1-10<br>10-100	0.9	[32]
Co(II)TAPc-TA/Au	DPV	7.4	10-100	0.28	[33]
N-G/CoPc/GCE	Amperometry	0.1 M NaOH	1-8000	0.6	[34]
RuHCF/rGO/PIGE	SWV	5	5.12-25.58	1.7	[35]
**PCs/CPE**	**Amperometry**	**4**	**5-10000**	**1**	**This work**

Table S5. Determination of GSH spiked in human serum by successive additions.

Added (μM)	Expected (μM)	Recovery (%)	RSD (%)
20	19.2	96	4.2
40	39.6	99	2.5
60	54.9	91.5	1.7

Table S6. Determination of GSH spiked in human serum by a non-successive addition method.

Added (μM)	Detected (μM)	Recovery (%)	RSD (%)
10	10.7	107	3.0
20	18.9	94.5	2.4
50	46	92	1.1

References

1. L. Wang, Q.Q. Teng, X.T. Sun, Y.T Chen, Y.M, Wang, H. Wang, Y.F. Zhang, Facile synthesis of metal-organic frameworks/ordered mesoporous carbon composites with enhanced electrocatalytic ability for hydrazine, J. Colloid. Interf. Sci. 512(2018) 127-133.
2. L. Wang, T.J. Meng, H.X. Jia, Y. Feng, T. Gong, H. Wang, Y.F. Zhang, Electrochemical study of hydrazine oxidation by leaf-shaped copper oxide loaded on highly ordered mesoporous carbon composite, J Colloid Interface Sci. 549(2019) 98-104.
3. M. Gharani, A. Bahari, S. Ghasemi, Preparation of MoS_2-reduced graphene oxide/Au nanohybrid for electrochemical sensing of hydrazine, J. Mater. Sci, Mater Electron. 32(2021) 7765-7777.
4. Y.Q. Yao, X.H. Han, X.H Yang, J. Zhao, C.P Chai, Detection of Hydrazine at MXene/ZIF-8 Nanocomposite Modified Electrode, Chin. J. Chem. 39(2021) 330-336.
5. X.J. Zhang, J.B. Zheng, Amperometric hydrazine sensor based on the use of a gold nanoparticle-modified nanocomposite consisting of porous polydopamine, multiwalled carbon nanotubes and reduced graphene oxide, Mikrochim. Acta. 187(2020) 89.
6. N. Vishnu, A.S. Kumar, S. Badhulika, Selective in-situ derivatization of intrinsic nickel to nickel hexacyanoferrate on carbon nanotube and its application for electrochemical sensing of hydrazine, J. Electroanal. Chem. 837(2019) 60-66.
7. S. Li, W.S. Feng, X.H. Gao, A.M. Guo, H.J Li, Copper-based materials derived from metal-organic frameworks for electrochemical sensing of hydrazine, Micro Nano Lett. 16(2021) 478-483.
8. F. Asadi, S.N. Azizi, S. Ghasemi, Preparation of Ag nanoparticles on nano cobalt-based metal organic framework (ZIF-67) as catalyst support for electrochemical determination of hydrazine, J. Mater. Sci. Mater. Electron. 30(2019) 5410-5420.
9. A. Avanes, M. Hasanzadeh-Karamjavan, G. Shokri-Jarcheloo, Electrocatalytic oxidation and amperometric determination of hydrazine using a carbon paste electrode modified with beta-nickel hydroxide nanoplatelets, Mikrochim Acta, 186(2019) 441.

10. C.Q. Duan, Y.H. Dong, Q.L. Sheng, J.B. Zheng, A high-performance non-enzymatic electrochemical hydrazine sensor based on $NiCo_2S_4$ porous sphere, Talanta. 198(2019) 23-29.
11. S. Ghasemi, S.R. Hosseini, F. Hasanpoor, S. Nabipour, Amperometric hydrazine sensor based on the use of Pt-Pd nanoparticles placed on reduced graphene oxide nanosheets, Mikrochim Acta. 186(2019) 601.
12. A. Maleki, R. Rezaee, H. Daraei, B. Shahmoradi, N. Amini, Fabrication of a sensitive electrochemical sensor to environmental pollutant of hydrazine in real water samples based on synergistic catalysis of Ag@C core–shell and polyalizarin yellow R, J. Alloys Compd. 763(2018) 997-1004.
13. M.M. Shahid, P. Rameshkumar, W.J. Basirunc, U. Wijayantha, W.S. Chiu, P.S. Khiew, N.M. Huang, An electrochemical sensing platform of cobalt oxide@gold nanocubes interleaved reduced graphene oxide for the selective determination of hydrazine, Electrochim. Acta. 259(2018) 606-616.
14. A. Mousavi-Majd, S. Ghasemi, S.R. Hosseini, Zeolitic imidazolate framework derived porous ZnO/Co_3O_4 incorporated with gold nanoparticles as ternary nanohybrid for determination of hydrazine, J. Alloys Compd. 896(2022).
15. S. Vinoth, P. Sampathkumar, K. Giribabu, A. Pandikumar, Ultrasonically assisted synthesis of barium stannate incorporated graphitic carbon nitride nanocomposite and its analytical performance in electrochemical sensing of 4-nitrophenol, Ultrasonics Sonochemistry, Ultrason. Sonochem. 62(2020) 104855.
16. Y.Y. Zhou, J. Zhao, S.H. Li, M.J. Guo, Z. Fan, An electrochemical sensor for the detection of p-nitrophenol based on a cyclodextrin-decorated gold nanoparticle-mesoporous carbon hybrid, Analyst. 144(2019) 4400-4406.
17. A.A. Ansari, M. Alam, M.A. Ali, Nanostructured CeO_2: Ag platform for electrochemically sensitive detection of nitrophenol, Colloids Surf. A. 613(2021) 126116.
18. R. Suresh, K. Giribabu, R. Manigandan, S. Praveen Kumar, S. Munusamy, S. Muthamizh, V. Narayanan, Polyaniline Nanorods: Synthesis, Characterization, and Application for the Determination ofpara-Nitrophenol, Anal. Lett. 49(2015) 269-281.
19. M.M. Alam, A.M. Asiri, M.M. Rahman, Electrochemical detection of 2-Nitrophenol using a glassy carbon electrode modified with BaO Nanorods, Chem. Asian. J. 16(2021) 1475-1485.
20. K. Nejati, K. Asadpour-Zeynali, Z. Rezvani, R. Peyghami, Determination of 2-nitrophenol by electrochemical synthesized Mg/Fe layered double hydroxide sensor, Int. J. Electrochem. Sci. 9(2014) 5222-5234.
21. H.S. Yin, Y.L. Zhou, S.Y. Ai, L. Cui, L.S. Zhu, Electrochemical determination of 2-Nitrophenol in Water Samples using Mg-Al-SDS Hydrotalcite-Like Clay modified glassy carbon electrode, Electroanalysis. 22(2010) 1136-1142.
22. J.H. Li, L.Z. He, J.B. Jiang, Z.F. Xu, M.Q. Liu, X. Liu, H.X. Tong, Z. Liu, D. Qian, Facile syntheses of bimetallic Prussian blue analogues ($K_xM[Fe(CN)_6]\bullet nH_2O$, M=Ni, Co, and Mn) for electrochemical determination of toxic 2-nitrophenol, Electrochim Acta, 353(2020) 136579.
23. L. Chu, L. Han, X.L. Zhang, Electrochemical simultaneous determination of nitrophenol isomers at nano-gold modified glassy carbon electrode, J. Appl. Electrochem. 41(2011) 687-694.
24. J.J. Zhang, S.Q. Cui, Y.P. Ding, X.X. Yang, K. Guo, J.T. Zhao, Bioelectronics, Two-dimensional mesoporous $ZnCo_2O_4$ nanosheets as a novel electrocatalyst for detection of o-nitrophenol and p-nitrophenol, Biosens. Bioelectron. 112(2018) 177-185.
25. B.M. Hryniewicz, E.S. Orth, M. Vidotti, Enzymeless PEDOT-based electrochemical sensor for the detection of nitrophenols and organophosphates, Sens. Actuators B Chem. 257(2018) 570-578.
26. B.Q. Yuan, X.Y. Zeng, C.Y. Xu, L. Liu, Y.H. Ma, D.J. Zhang, Y. Fan, Electrochemical modification of graphene oxide bearing different types of oxygen functional species for the electro-catalytic oxidation of reduced glutathione, Sens. Actuators B Chem. 184(2013) 15-20.
27. W.T. Wu, X. Chen, Y.T. Jiao, W.T. Fan, Y.L. Liu, W.H. Huang, Versatile Construction of Biomimetic Nanosensors for Electrochemical Monitoring of Intracellular Glutathione, Electroanal. Chem. 134(2022) e202115820.
28. L.Z. Zhao, L. Zhao, Y. Miao, C.X. Zhang, Selective electrochemical determination of glutathione from the leakage of intracellular GSH contents in HeLa cells following doxorubicin-induced cell apoptosis, Electrochim Acta. 206(2016) 86-98.
29. P. Lee, R.G. Compton, Selective electrochemical detection of thiol biomarkers in saliva using multiwalled carbon nanotube screen-printed electrodes, Sens. Actuators B Chem. 209(2015) 983-988.
30. P.T. Lee, L.M. Goncalves, R.G. Compton, Electrochemical determination of free and total glutathione in human saliva samples, Sens. Actuators B Chem. 221(2015) 962-968.
31. B.Q. Yuan, R.C. Zhang, X.X. Jiao, J. Li, H.Z. Shi, D.J. Zhang, Amperometric determination of reduced glutathione with a new Co-based metal-organic coordination polymer modified electrode, Electrochem. Commun. 40(2014) 92-95.
32. S. Kannappan, L. Ramachandra Bhat, N. Nesakumar, K.J. Babu, A.J. Kulandaisamy, J.B.B Rayappan, Design and development of a non-enzymatic electrochemical biosensor for the detection of Glutathione, Electroanalysis. 34(2022)1-12.
33. M.N. Abbas, A.A. Saeed, M.B. Ali, A. Errachid, N. Zine, A. Baraket, B. Singh, Biosensor for the oxidative stress biomarker glutathione based on SAM of cobalt phthalocyanine on a thioctic acid modified gold electrode, J. Solid State Electrochem. 23(2019) 1129-1144.
34. H.Y. Xu, J.J. Xiao, B. Liu, S. Griveau, F. Bedioui, Enhanced electrochemical sensing of thiols based on cobalt phthalocyanine immobilized on nitrogen-doped graphene, Biosens. Bioelectron. 66(2015) 438-444.
35. S. Saranya, B. Geetha, P.N. Deepa, Simultaneous detection of glutathione, threonine, and glycine at electrodeposited RuHCF/rGO–modified electrode, Ionics. 25(2019) 5537-5550.
